# A Comprehensive Model for Gambling Behaviors: Assessment of the Factors that can Contribute to the Vulnerability and Maintenance of Gambling Disorder

**DOI:** 10.1007/s10899-021-10024-3

**Published:** 2021-04-12

**Authors:** Alessio Gori, Eleonora Topino, Giuseppe Craparo, Ilaria Bagnoli, Vincenzo Caretti, Adriano Schimmenti

**Affiliations:** 1 Department of Health Sciences, University of Florence, Piazza S. Marco 4, 50121 Firenze, Italy; 2 Department of Human Sciences, LUMSA University of Rome, Via della Traspontina, 21, 00193 Rome, Italy; 3 Faculty of Human and Social Sciences, UKE—Kore University of Enna, Cittadella Universitaria, 94100 Enna, Italy

**Keywords:** Gambling disorder, Vulnerability, Maintenance, Craving, Behavioural addiction

## Abstract

Gambling Disorder is a complex and multifaceted phenomenon which requires a careful understanding by analysing both the life experiences and the psychopathological components linked to this type of behaviour. This study aimed to apply a Comprehensive Model of Addiction and to delve deeper the dimensions involved in the vulnerability and maintenance of the disease. Therefore, the effect of alexithymia and traumatic experiences in mediating the relationship between insecure attachment and dissociation, as well as the roles of impulsiveness and compulsiveness in influencing obsessiveness were explored in pathological gamblers. A sample composed of 253 individuals with a mean age of 47.8 years (*SD* = 12.4) with a diagnosis of Gambling Disorder (82.6% males, 17.4% females) completed the battery of measures. Results showed that alexithymia significantly mediates the relationship between insecure attachment and dissociation, while no significance was found in the effect of complex trauma. Furthermore, a significant impact role of impulsiveness and compulsiveness in determining obsessiveness was found. Therefore, the data suggested that alexithymia may increase the risk of developing a gambling disorder, mediating the association between insecure attachment and dissociation. The model of craving which could have a core role in disease maintenance processes was also confirmed, highlighting a significant influence of impulsiveness and compulsiveness on obsessiveness. Such findings might have relevant implications to increase the effectiveness of both preventive interventions and therapeutic works, favouring positive results for a better mental health of the subjects.

## Introduction

Gambling Disorder (GD) is a “behavioural addiction” delineated as a “*persistent and recurrent problematic gambling behavior leading to clinically significant impairment or distress*” (American Psychiatric Association, 2013, p. 585), and represents a relevant public health concern (Potenza et al., 2019). According to Stewart and Zack (2008) and Dechant (2014), gambling could be linked to several motivations, such as the social and the financial ones, enhancement or coping. Concerning to the last two, the authors referred, on the one hand, to the search for strong emotions and the tendency to use gambling to experience adrenaline and to face boredom (Lam, 2007; Lee et al., 2007; Lesieur, 2001; Neighbors et al, 2002), on the other, to attempt to find a temporary escape from negative feelings in this activity, such as depression and anxiety (Barrault et al., 2019; Weatherly & Cookman, 2014). In other words, gambling could become an emotions regulator and the acquisition of this function determines the transition from recreation to psychopathology (Barrada et al., 2019; Grant et al., 2012; Wardell et al., 2015; Zakiniaeiz et al., 2017).

These aspects appear consistent with the theoretical conceptualization of addiction by Caretti and colleagues (2018), who grouped and integrated some evidence of the field reported in the scientific literature and identified some variables that could have a key role in addiction disorders. In more detail, addictive behaviors are considered the result of interactions between insecure attachment, emotional dysregulation, complex trauma, dissociation, impulsiveness, compulsiveness, and obsessiveness. Indeed, a broad line of research highlighted the key role of a negative individual developmental environment as risk factor for addictive behaviours (Flores, 2004; Schimmenti et al., 2014). Specifically, an insecure attachment may lead to a deficiency in emotional regulation skills (Beebe & Lachmann, 2002). Parallelly, a pathological developmental environment could be a source of traumatic experiences during childhood, such as neglect, abuse, violence (Schimmenti, 2018), for which the lack of emotional regulation may increase the difficulty in effectively coping. All this may lead to a tendency to use dissociative responses, such as those linked to the addiction, to face aversive situations and alleviate painful emotions (Craparo et al., 2014; Evren et al., 2013; Schimmenti, 2016). Moreover, impulsiveness, compulsiveness and obsessiveness were identified as central dimensions in craving phenomenon (Caretti et al., 2016): indeed, the first two refer to an overwhelming search for pleasure and the reduction of discomfort, respectively (Perales et al., 2020; Quinn & Harden, 2013), leading to an obsessive attitude toward the addiction object (MacKillop et al. 2006). In other words, craving drives the search for immediate gratification of dysregulated impulses and allows the individual to tolerate, in short, otherwise painful affective states, making the object of dependence central in the subject's life with recurring thoughts and images and thus facilitating the perpetuation of the behaviours linked to the disease (Caretti et al., 2010).

On that bases, the present research aim was to verify and deepen the association between the variables involved in the theoretical conceptualization of addiction by Caretti and collagues (2018) in pathological gamblers.

Indeed, Gambling Disorder is a severe condition which affects the economic, occupational, relational, familiar and psychological areas of life of the pathological gamblers (Derevensky, 2007; Edgerton et al., 2015), sometimes also leading to legal problems and suicidal behaviour (Hartmann & Blaszczynski, 2018). Several correspondences regarding neurobiological evidence, comorbidity, symptomatic behaviour, susceptibility to treatment and aspects of the course, motivated the DSM-5 task force to move GD in the section of addiction disorders (Hasin et al., 2013), confirming that the addictive propensity may develop both from substances and from behaviours (Caretti et al., 2018; Perales et al., 2020). In this regard, several studies showed that gambling behaviours may represent an external regulator of internal emotional states (Di Trani et al., 2017; Gori et al., 2016; Pace et al., 2015; Rogier & Velotti, 2018) and it correlated significantly with traumatic experiences (Hodgins et al., 2010; Lane et al., 2016), insecure attachment (Sherrer et al., 2007) and psychopathological traits, such as alexithymia (Bibby, 2016; Gori et al., 2016; Iraci-Sareri & Gori, 2012; Maniaci et al., 2015) and dissociation (Craparo et al., 2015; Gori et al., 2016; Griffiths et al., 2006; Schluter & Hodgins, 2019; Williams et al., 2012). On the other hand, other researchers suggested the presence of high levels of impulsiveness, compulsiveness and obsessiveness in pathological gamblers (El‐Guebaly et al., 2012; Chowdhury et al., 2017; Okechukwu, 2019; Steel & Blaszczynski, 2002), supporting the views which consider craving as a construct of central importance in the maintenance and exacerbation of gambling disorder (Blaszczynski & Nower, 2002; Brevers & Noël, 2013; Sharpe, 2002), but also in the difficulty of treatment and the tendency to relapse (Oei & Gordon, 2008; Smith et al., 2010).

Given this framework, the present study aimed to expand and apply the theoretical implications of Caretti and collagues (2018) and the evidence of previous research converging in it, by elaborating a new Comprehensive Model of Addiction, in which two models including the factors that scientific literature suggests may have a core role in the development and maintenance of addictions have been outlined. Therefore, two models were hypothesized: in the first one, the Vulnerability model, the mediation roles of Alexithymia and Complex Trauma in the relationships between Insecure Attachment and Dissociation were explored, according to the Vulnerability Model; while in the second one, the roles of Impulsiveness and Compulsiveness in affecting Obsessiveness were analyzed, determining the Craving Model.

## Method

### Participants and Procedure

The study involved 253 individuals who have been diagnosed with a Gambling Disorder (82.6% males, 17.4% females) and with a mean age of 47.8 years (*SD* = 12.4). All participants were recruited in collaboration with the National Health Service (NHS) and several private institutions for the treatment of Gambling Disorder in various Italian Regions. A cross-sectional design was adopted for this study. The inclusion criteria were a diagnosis of Gambling Disorder according to DSM-5, minimum age of 18 years, and good knowledge of the Italian language, while all the subjects with dual diagnosis were excluded. Each participant was undergoing inpatient/outpatient therapy in the recruitment center and filled in the paper–pencil questionnaires with the help of the research assistants in a one-to-one setting, for approximately 40 min. In the sample, 21.7% of the participants were unemployed, 16.2% of them were employees, and the 15.0% were retired; 40.7% were married and 28.1% were single. Regarding qualifications, 45.6% declared that they had a lower secondary school diploma and another 31.2% reported to have graduated high school; 5.5% of the sample said they only attended primary school while 4.0% said they had a bachelor’s degree or a master’s degree (see Table 1). The measures were collected anonymously after all the participants were informed about the aim of the research and gave written informed consent in accordance with the Declaration of Helsinki (World Medical Association, 2013). The subjects also completed a demographic questionnaire (i.e., age, sex, weight, height) and they were told that could leave the study any time and that they would not be receing any form of payment for participating in the study.

**Table 1:** Demographic characteristics of the sample (n = 253)

Characteristics		M ± SD	n	%
	Age	47.8 ± 12.4		
Sex				
	Males		209	82.6
	Females		44	17.4
Marital Status				
	Single		71	28.1
	Married		103	40.7
	Cohabiting		8	3.2
	Separated		14	5.5
	Divorced		10	4.0
	Widowed		10	4.0
	Missing Values		37	14.6
Education				
	Elementary school (5 years)		14	5.5
	Middle School diploma (8 years)		115	45.6
	High School diploma (13 years)		79	31.2
	University degree (16 years)		2	.8
	Master’s degree (18 years)		8	3.2
	Missing values		35	13.8
Professional Condition				
	Unemployed		55	21.7
	Looking for first job		4	1.6
	Entrepreneur		6	2.4
	Employee		41	16.2
	Artisan		20	7.9
	Trader		6	2.4
	Armed forces		4	1.6
	Student		4	1.6
	Retired		38	15.0
	Other		36	14.2
	Missing values		39	15.4

### Measures

#### Psychological Treatment Inventory—Attachment Styles Scale (PTI-ASS)

The Psychological Treatment Inventory—Attachment Styles Scale (PTI-ASS; Giannini et al., 2011) is a section of the Psychological Treatment Inventory (Gori et al., 2015) designed to explore the quality of romantic relationships and the correlated behaviors, emotions and thoughts. These components are evaluated with 22 items on a 5-point Likert scale (from 1 = “Not at All” to 5 = “A Great Deal”) and this allow to assess attachment style considering the categories of secure (comfort in closeness with the partner and absence of fear of abandonment), preoccupied (fear of abandonment, with constant concern about their relationship and a desperately need intimacy), avoidant (discomfort in closeness, dependence and little emotional investment in relationships), and unresolved (fear and discomfort in intimacy, despite the desire to have emotionally close relationships). The subscales’ Cronbach α in the current study were of 0.80, 0.80, 0.73 and 0.66, respectively.

#### Twenty-Items Toronto Alexithymia Scale (TAS-20)

The Twenty-Items Toronto Alexithymia Scale (TAS-20; Bagby et al., 1994a; Bagby et al., 1994b) is a self-report scale designed to assess the level of alexithymia. It consists of three factors, evaluated with 20 items on a 5-point Likert scale (from 1 = “strongly disagree” to 5 = “strongly agree”): 1) difficulty in identifying feelings and distinguishing between feelings and bodily sensations in emotional activation; 2) difficulty in the verbal expression of emotions; 3) externally oriented thinking. It is possible to calculate both the subscales scores and the total alexithymia score. This latter has a cut-off of 61: above this value the scale indicates an alexithymic condition. Scores equal or less than 51 indicate non alexithymia, and scores of 52 to 60 detect a possibility of alexithymia. In this sample the Italian versions of the TAS-20 (Bressi et al., 1996) was used and showed a good internal consistency (Cronbach α = 0.78).

#### Traumatic Experiences Checklist (TEC)

The Traumatic Experiences Checklist (TEC; Nijenhuis et al., 2002) is a self-report measure designed to take over the presence and the impact of potentially traumatizing events in the subjects’ life histories. It consists of 29 types of potentially traumatizing events (such as emotional neglect, emotional abuse, physical abuse, sexual harassment, sexual abuse and bodily torea) scored on a true–false form. Furthermore, it is asked to rate the extent of the impact for the events that occurred on a 5-point Likert scale (1 = “none”, 2 = “a little bit”, 3 = “a moderate amount”, 4 = “quite a bit”, 5 = “an extreme amount”). In this study, the Italian version of the TEC (Schimmenti, 2018; Cronbach α in the present research of 0.78) was used to assess the impact of traumatic events by summing the scores of the Likert scale for each potentially traumatizing event occurred.

#### Dissociative Experience Scale-II (DES-II)

The Dissociative Experiences Scale II (DES-II; Carlson & Putnam, 1993) is a self-report scale designed to measure a variety of types of dissociation. It is a valid screening tools for dissociative disorders and consists of 28 items, ranged from 0%, or “never,” to 100%, “always”, in which the rate of occurring of various dissociative experiences in subjects’ daily life is asked: higher scores indicate greater levels of psychological dissociation. In addition to the total score, this tool provides three subscales which could be useful to better define the dissociative condition: 1) dissociative amnesia, which measures memory loss; 2) absorption and imaginative involvement, which measures the level of absorption on internal or external cues; 3) depersonalization-derealization, which measures feeling detached and sense of unreality from one’s self or the world. In this study, the Italian version of the DES-II was used (Schimmenti, 2016; Cronbach’s α in the current research of 0.94).

#### Barratt Impulsiveness Scale (BIS-11)

The Barratt Impulsiveness Scale (BIS-11; Patton et al., 1995) is a self-repot measure designed to assess general impulsiveness. It consists of 30 items on a 4-point Likert Scale (1 = rarely/never, 4 = almost always/always) which form six first-order factors (attention, motor, self-control, cognitive complexity, perseverance, cognitive instability) grouped into three second-order factors: 1) Attentional Impulsiveness, composed by the first-order factors attention and cognitive instability; 2) Motor Impulsiveness, consisting of the first-order factors motor and perseverance; 3) Non-Planning impulsiveness, which includes the first-order factors complexity and self-control. The total score is achieved by adding the first or second order factors and higher scores indicate greater levels of impulsivity. In the present study the Italian version of Fossati and colleagues (2001) was used, showing a good internal consistency (α = 0.72).

#### Yale-Brown Obsessive Compulsive Scale- Second Edition (Y-BOCS-II)

The Yale-Brown Obsessive Compulsive Scale- Second Edition (Y-BOCS-II; Storch et al., 2010) is a measure designed to assess obsessive–compulsive disorder (OCD) symptoms severity and type. It consists of the Symptom Checklist (a list of possible obsessions, compulsions, and avoidance behaviours experienced over the past 30 days) and the Severity Scale. This latter is composed by 10 items for evaluating impairment and severity of obsessions and compulsive behaviours (5 item for each one) in a 6-point Likert scale (from 0 = none; to 5 = extreme), basing on five dimensions (time/frequency, interference, distress, resistance, and degree of control). The scores of both obsessive and compulsive symptoms can range from 0 to 25 and the total score from ranges from 0 to 50. In the present study the Italian version of was used (Melli et al., 2015), which showed a Cronbach’s alpha of 0.94 for the total scale and 0.94 and 0.95 for the two subscales.

### Data Analysis

All the statistical analyses were performed using the software SPSS 25.0 for Windows. Descriptive statistics for the sample and measures were calculated. Then, Pearson’s *r* correlations were used to analyse the associations between the variables. Moreover, the two explorative models (Vulnerability and Craving) were tested to assess the relationship among the factors linked to the disease. First, mediation analyses were therefore performed to verify the hypothesized relationships, by using model 4 in the macro-program PROCESS 3.4 (Hayes, 2018). To verify the significance of the indirect effect, two different procedures were implemented: the bootstrapping technique for each of 5,000 bootstrapped samples with the 95% of confidence interval, and the Monte Carlo method (using the MEDIATE macro program; Hayes & Preacher, 2014) with the same bootstrap parameters. Finally, multiple regression analysis was applied to derive craving model which is allow to predict Obsessiveness (the dependent variable) from Impulsiveness and Compulsiveness (the independents variables). In this case, to test the Type 1 error, Bias corrected accelerated (BCa) bootstrapping based on 5,000 samples with the 95% of confidence interval was applied.

## Results

Descriptive statistics of the sample and the measures are reported in Tables 1 and 2, respectively.

**Table 2:** Correlations matrix, mean and standard deviations of the measures

	PTI(SEC)	PTI(PRE)	PTI(AVO)	PTI(UNR)	TEC	TAS20	DES-II	BIS-11	YOSS	YCOM
PTI(SEC)	1	− .012	− .107	− .296^**^	.038	− .504^**^	− .276^**^	− .294^**^	− .087	− .091
PTI(PRE)		1	− .364^**^	.368^**^	.131	.282^**^	.152^*^	.007	.196^**^	.168^*^
PTI(AVO)			1	− .121	.070	.042	.080	.217^**^	.044	− .042
PTI(UNR)				1	.050	.252^**^	.322^**^	.092	.044	.000
TEC					1	− .025	.102	− .081	.078	.117
TAS20						1	.385^**^	.479^**^	.248^**^	.144^*^
DES − II							1	.298^**^	.354^**^	.355^**^
BIS − 11								1	.297^**^	.234^**^
YOSS									1	.547^**^
YCOM										1
*M*	15.33	11.49	10.73	7.23	6.70	52.56	11.65	71.87	4.63	4.38
*SD*	4.20	4.66	3.88	2.46	6.03	13.78	10.87	11.99	5.02	5.03

Bold values indicate significant correlations

**Correlation is significant at the 0.01 level (2-tailed)

*Correlation is significant at the 0.05 level (2-tailed)

PTI(SEC), Secure attachment style (PTI-ASS); PTI(PRE), Preoccupied attachment style (PTI-ASS); PTI(AVO), Avoidant attachment style (PTI-ASS); PTI(UNR), Unresolved attachment style (PTI-ASS); TEC, Traumatic Experiences Checklist; TAS20, Twenty-Items Toronto Alexithymia; DES-II, issociative Experiences Scale II; BIS11, Barratt Impulsiveness Scale 11; YOBS, Impulsiveness (Y-BOCS-II); YCOM, Compulsiveness (Y-BOCS-II)

Significant associations emerged among some variables included in this study, as showed in Table 2.

**Fig. 1:**
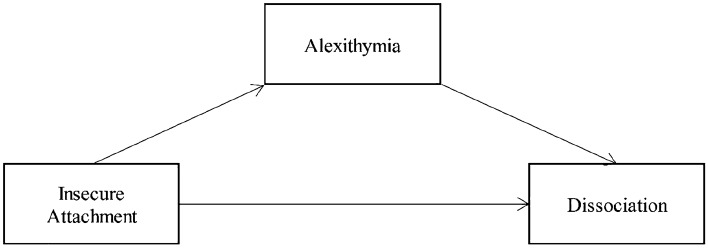
Relationship between Insecure Attachment and Dissociation, with Alexithymia as a mediator

Furthermore, a mediation model was performed to investigate the possible mediator role of Complex Trauma between Insecure Attachment and Dissociation. As suggested by the absence of significant correlations, this relation was not confirmed, with insignificant path both from Insecure Attachment to Complex Trauma (*β* = 0.050, *p* = 0.625) and from Complex Trauma to Dissociation (*β* = 0.086, *p* = 0.376). Then, it was hypothesized that Alexithymia could mediate the causal relationship of Insecure Attachment on Dissociation (see Fig. 1). Results supported this hypothesis (see Table 3).

**Table 3:** Mediation model coefficients

Consequent								
Antecedent		M (Alexithymia)				Y (Dissociation)		
		Coeff	SE	*p*		Coeff	SE	*p*
X (InsecureAttachment)	*a*	1.528	0.401	< 0.001	*c’*	0.920	0.271	< 0.001
M (Alexithymia)		–	–	–	*b*	0.262	0.047	< 0.001
Constant	*i*_*M*_	40.221	3.087	< 0.001	*i*_*Y*_	− 10.175	2.758	< 0.001
		*R*^*2*^ = 0.070				*R*^*2*^ = 0.229		
		*F*(1, 192) = 14.525, *p* < .001				*F*(2, 191) = 28.321, *p* < .000		

Insecure Attachment demonstrated a significant positive influence on Dissociation (estimating the path *c*, *β* = 0.322, *p* < 0.001) and affected it indirectly through Alexithymia. Indeed, Insecure Attachment was associated with Alexithymia (*β* = 0.265, *p* < 0.001), the mediator variable (estimating and testing the path *a* in Table 3), which in turn showed an effect on Dissociation (*β* = 0.327, *p* < 0.001), estimating the path *b* in Table 3. So, the effect of Insecure Attachment on Dissociation was reduced after controlling Alexithymia (path *c’* in Table 3), but it still remained significant (*β* = 0.224, *p* < 0.001). In Table 4, model effects indices and path were summarized.

**Table 4:** Model effect indices

Total Effect	Direct Effect	Indirect Effect	Partial Standardized Indirect Effect	Completely Standardized Indirect Effect	95% CI indirect effect	
					Percentile Bootstrap	Monte Carlo
1.32	0.92	0.40	0.04	0.10	[0.198, 0.336]	[0.173, 0.669]

**Fig. 2:**
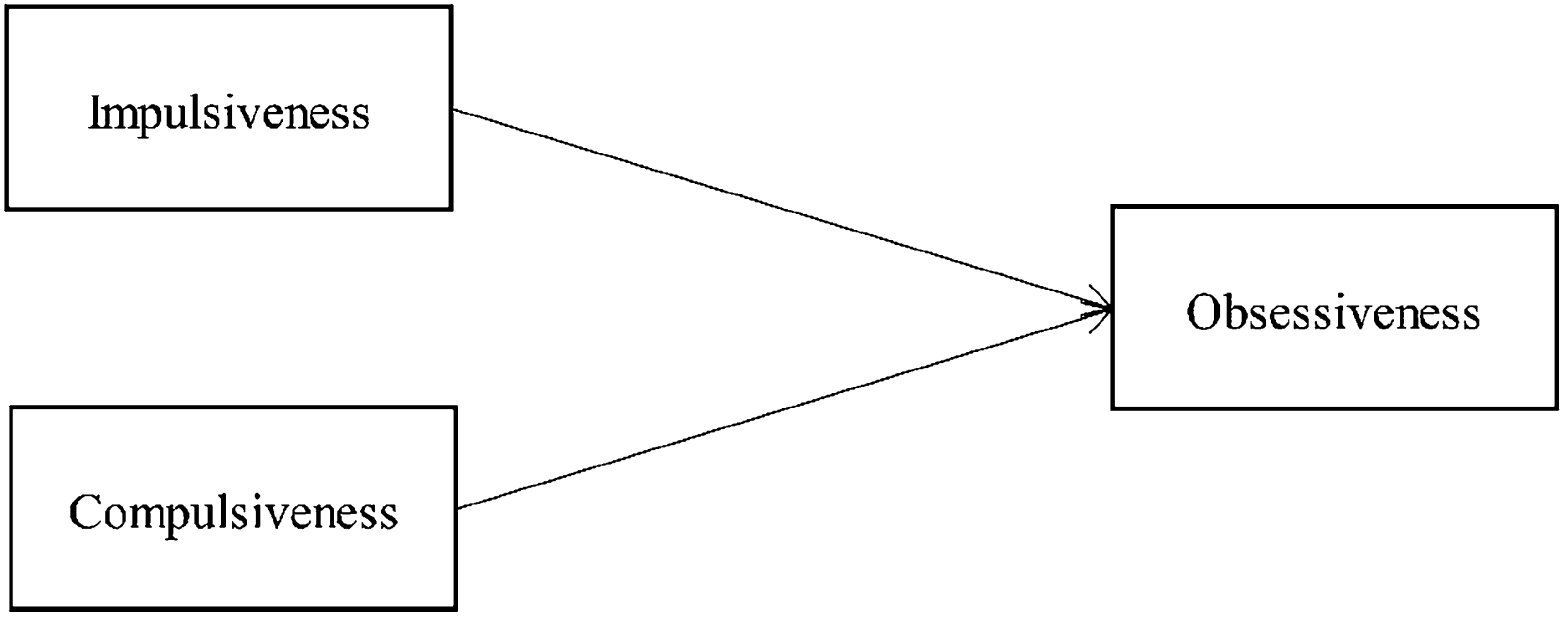
The impact of Impulsiveness and Compulsiveness in predicting Obsessiveness (Craving Model)

Concerning the indirect effect, the bootstrapping procedure (Boot LLCI = 0.198—Boot ULCI = 0.336) and the Monte Carlo Method (LLCI = 0.173—ULCI = 0.669) showed its significance (see Table 4). Then, a multiple linear regression was calculated to predict Obsessiveness based on Impulsiveness and Compulsiveness (the Craving Model). A significant regression equation was found (*F* (2197) = 48.398; *p* < 0.001), with an *R*^*2*^ of 0.329: the independent variables explained a significant percentage of the variance (33%). The analysis showed that both impulsiveness (*β* = 0.179, *p* < 0.01) and compulsiveness (*β* = 0.505, *p* < 0.001) are significant predictors of obsessiveness (see Table 5 and Fig. 2), Finally, both the effects of impulsiveness and compulsiveness on obsessiveness were confirmed by the Bias corrected accelerated (BCa) bootstrapping (Boot LLCI = 0.037- Boot ULCI = 0.119; Boot LLCI = 0.363—Boot ULCI = 0.642, respectively).

**Table 5:** Multiple linear regression predicting Obsessiveness (Craving Model)

Coefficients^a^								
		Unstandardized Coefficients		Standardized Coefficients	*t*	*p*	Unstandardized Coefficients^b^	
		B	Std. Error	Beta			*p* (2-tailed)	BCa 95% Confidence Interval
1	(Constant)	− 3.044	1.816		− 1.676	0.095	0.034	[− 5.832, − .318]
	BIS11	0.076	0.026	0.179	2.979	0.003	0.000	[.037, .119]
	YCOM	0.504	0.060	0.505	8.420	0.000	0.000	[.363, .642]

^a^Dependent Variable: YOBS

^b^Bootstrap results are based on 5000 bootstrap samples

BIS11, Barratt Impulsiveness Scale 11; YOBS, Obsessiveness (Y-BOCS-II); YCOM,  Compulsiveness (Y-BOCS-II)

## Discussion

Based on the dimensions identified in the theoretical perspective of addiction by Caretti and colleagues (2018), the present research aimed to investigate the relationship between several factors that may be related to the condition of Gambling Disorder. Therefore, a Comprehensive Model of Addiction was elaborated, which includes the variables that may be involved in the development or maintenance of the disease. Specifically, the role of Insecure Attachment, Alexithymia and Complex Trauma in contributing to dissociation and that of Impulsiveness and Compulsiveness in affecting Obsessiveness were explored, outlining the Vulnerability and Craving models, respectively.

Results confirmed the mediation effect of Alexithymia in the relationship between Insecure Attachment and Dissociation. So, experiences of neglect and the absence of emotional reciprocity typical of an insecure attachment compromise the development of adequate skills of both self and interactive affects regulation (Bowlby, 1969): this will hinder the use of functional strategies to cope with distressing conditions (Morris et al., 2007) in a relatively stable and long-lasting way throughout the life of the individual (Bowlby, 1988; Collins & Read, 1994; Hazan & Shaver, 1994). To confirm this, several studies have found elevated levels of alexithymia in subjects with a gambling disorder (see, for a review, Marchetti et al., 2019) and an excessive tendency to use expressive suppression strategies (Rogier & Velotti, 2018), which limits the behavioral expression of malaise, without however increasing psychological well-being (Gross & John, 2003; John & Gross, 2004). Obviously, this does not eliminate the state of suffering: alexithymia is not the same of athymia (Taylor et al., 1997) and disordered gambler show high levels of anxiety and depressive symptomatology (Barrault et al., 2017; Marchica et al., 2019). In light of this, many studies interpreted gambling disorder activity as resorting to a dissociative state aimed at coping with conditions of psychological distress and affective dysregulation that the subject has not learned to manage effectively in his attachment relationship (Goldstein et al., 2018; Jacobs, 1986; McCormick et al., 2012; Rogier & Velotti, 2018; Tang et al., 2019). However, based on the dimensions considered by Caretti and colleagues (2018), the Vulnerability model of Addiction also provided for an impact role of Complex Trauma, which was not found in this research. This appears in contrast to previous findings (e.g., Hodgins et al., 2010), but can be understood in the light of the study by Green and colleagues (2016), in which it is reported how gambling severity is influenced not so much by the trauma per se, but by the symptoms of PTSD and hyperarousal deriving from it and that are however reported less in pathological gamblers, which tend to inhibit mechanisms of emotional expression. This may explain their lack of involvement in interactions with the other variables examined in the present study, although traumatic experiences appear relevant in facilitating a vulnerability to other forms of Addiction (Barrett & Turner, 2006; Dembo et al., 1988; Stewart, 1996; Widom et al., 1999). Concerning the Craving Model, the results confirmed the influence of impulsiveness and compulsiveness in obsessiveness. Supporting this, several studies have shown that high levels of pleasure-seeking impulses are related to gambling disorder (Blain et al., 2015; Cyders & Smith, 2008; Haw, 2017; Steward et al., 2017), while other research has highlighted how this activity can represent an avoidant coping strategy to contrast negative emotional states (Blaszcsynski & Nower 2002; Leeman & Potenza, 2012; James et al., 2016). Therefore, subjects choose immediate pleasure and relief in the short term, which however will lead to negative consequences, depressive symptoms, and problems in the medium and long term (Folkman & Moskowitz, 2000; Power et al., 2012; Tice et al., 2001), feeding the need to perpetuate this behaviour. Indeed, the interaction of craving variables and their role in maintaining addictive behaviour is evident in Loss-Chasing (i.e., the drive to continue gambling in an attempt to recover losses), which can be seen as a key feature of gambling disorder (Bibby, 2016).

This study also has some limitations that should be addressed. Firstly, the sample was mainly composed of men. This is in line with a real trend of gambling disorder, in which there is an over-representation of males (e.g., Shaffer & Hall, 2001). However, it could be interesting to analyse the specificity of problem gambling in women and the differences between them and men. Furthermore, no distinctions were made between the several types of gambling. The different types of activities (such as strategic or luck games) could imply differences in the psychological and psychopathological profile of pathological gamblers: so, could be important for future research re-propose the models presented in this study to subjects involved in different kind of gambling and check for any differences. Finally, the cross-sectional nature of this research and the absence of a control group do not allow for certain inferences about the causal/directional relationship between the variables. Future research should conduct longitudinal studies to permit this in order to lead to safer conclusions.

Despite the limitations, however, the value and innovativeness of this study concerns the elaboration of a new Comprehensive model of Addiction and its application to assess the peculiar dimensions involved in Gambling Disorders, as well as their relationships. This allows for an integration of the previous evidences and research of the field, by applying and expanding the theoretical perspective of Caretti and colleagues (2018), and highlighting the factors that may contribute to the vulnerability and maintenance of the disease. Therefore, this study could be a further step forward to enrich the discussion and ponder on the risk factors in the development and chronicity of addiction, that should be further investigated with future research.

## Conclusion

This study allows for a greater understanding of the variables that play a central role in determining vulnerability and in the maintenance of gambling disorder. In this way, a new interpretation of the problem is offered, and this could be functional to increase the effectiveness of both preventive interventions and therapeutic activity, favouring positive results to improve the mental health of the subjects.

## Data Availability

Research data are not shared.

## References

[CR1] American Psychiatric Association (2013). Diagnostic and statistical manual of mental disorders.

[CR2] Bagby RM, Parker JD, Taylor GJ (1994). The twenty-item Toronto Alexithymia Scale—I. Item selection and cross-validation of the factor structure. Journal of psychosomatic research.

[CR3] Bagby RM, Taylor GJ, Parker JD (1994). The twenty-item Toronto Alexithymia Scale—II Convergent, discriminant, and concurrent validity. Journal of psychosomatic research.

[CR4] Barrada JR, Navas JF, de Lara CMR, Billieux J, Devos G, Perales JC (2019). Reconsidering the roots, structure, and implications of gambling motives: An integrative approach. PLoS ONE.

[CR5] Barrault S, Bonnaire C, Herrmann F (2017). Anxiety, depression and emotion regulation among regular online poker players. Journal of gambling studies.

[CR6] Barrault S, Mathieu S, Brunault P, Varescon I (2019). Does gambling type moderate the links between problem gambling, emotion regulation, anxiety, depression and gambling motives. International Gambling Studies.

[CR7] Barrett AE, Turner RJ (2006). Family structure and substance use problems in adolescence and early adulthood: examining explanations for the relationship. Addiction.

[CR8] Beebe B, Lachmann F (2002). Organizing principles of interaction from infant research and the lifespan prediction of attachment: Application to adult treatment. Journal of Infant, Child, and Adolescent Psychotherapy.

[CR9] Bibby P (2016). Loss-Chasing, Alexithymia, and Impulsivity in a Gambling Task: Alexithymia as a Precursor to Loss-Chasing Behavior When Gambling. Frontiers in Psychology.

[CR10] Blain B, Richard Gill P, Teese R (2015). Predicting problem gambling in Australian adults using a multifaceted model of impulsivity. International Gambling Studies.

[CR11] Blaszczynski A, Nower L (2002). A pathways model of problem and pathological gambling. Addiction.

[CR12] Bowlby J (1969). Attachment and loss.

[CR13] Bowlby J (1988). A secure base: parent-child attachment and healthy human development.

[CR14] Bressi C, Taylor G, Parker J, Bressi S, Brambilla V, Aguglia E, Allegranti I, Bongiorno A, Gilberti F, Bucca M, Todarello O, Callegari C, Vender S, Gala C, Invernizzi G (1996). Cross validation of the factor structure of the 20-item Toronto Alexithymia Scale: an Italian multicenter study. Journal of psychosomatic research.

[CR15] Brevers D, Noël X (2013). Pathological gambling and the loss of willpower: a neurocognitive perspective. Socioaffective neuroscience & psychology.

[CR16] Caretti, V., Craparo, G., Giannini, M., Gori, A., Iraci-Sareri, G., Lucchini, A., Rusignuolo, I., & Schimmenti, A. (2016). *Addictive Behavior Questionnaire (ABQ)*. Manuale e questionari [Addictive Behavior Questionnaire (ABQ). Manual and questionnaires]. Firenze: Hogrefe editore.

[CR17] Caretti V, Craparo G, Schimmenti A, Caretti V, La Barbera D (2010). Developmental-relational factors of addiction. Addiction Biological and research aspects.

[CR18] Caretti V, Gori A, Craparo G, Giannini M, Iraci-Sareri G, Schimmenti A (2018). A new measure for assessing substance-related and addictive disorders: the addictive behavior questionnaire (ABQ). Journal of Clinical Medicine.

[CR19] Carlson, E. B., & Putnam, F. W. (1993). An update on the dissociative experiences scale. *Dissociation: progress in the dissociative disorders*.

[CR20] Chowdhury NS, Livesey EJ, Blaszczynski A, Harris AJ (2017). Pathological gambling and Motor Impulsivity: A Sistematic Review with Meta-Analysis. Journal of gambling Studies.

[CR21] Collins, N. L., & Read, S. J. (1994). Cognitive representations of attachment: The structure and function of working models.

[CR22] Craparo G, Ardino V, Gori A, Caretti V (2014). The Relationships between Early Trauma, Dissociation, and Alexithymia in Alcohol Addiction. Psychiatry Investigation.

[CR23] Craparo, G., Gori, A., Iraci,G., Pace, U. (2015) Personality and Clinical Dimensions of Pathological Gamblers. A pilot study. *Mediteranean Journal of Social Science,*6, p.612.

[CR24] Cyders MA, Smith GT (2008). Clarifying the role of personality dispositions in risk for increased gambling behavior. Personality and individual differences.

[CR25] Dechant K (2014). Show me the money: incorporating financial motives into the Gambling Motives Questionnaire. Journal of Gambling Studies.

[CR26] Dembo R, Dertke M, Borders S, Washburn M, Schmeidler J (1988). The relationship between physical and sexual abuse and tobacco, alcohol, and illicit drug use among youths in a juvenile detention center. International Journal of the addictions.

[CR27] Derevensky, J. L. (2007). Youth gambling and problem gambling: Another high risk behavior. *International Center for Youth Gambling and High-Risk Behaviors*, 1–14.

[CR28] Di Trani M, Renzi A, Vari C, Zavattini GC, Solano L (2017). Gambling Disorder and affect regulation: the role of alexithymia and attachment style. Journal of gambling studies.

[CR29] Edgerton JD, Melnyk TS, Roberts LW (2015). Problem gambling and the youth-to-adulthood transition: assessing problem gambling severity trajectories in a sample of young adults. Journal of gambling studies.

[CR30] El-Guebaly N, Mudry T, Zohar J, Tavares H, Potenza MN (2012). Compulsive features in behavioural addictions: the case of pathological gambling. Addiction.

[CR31] Evren C, Cınar O, Evren B, Ulku M, Karabulut V, Umut G (2013). The mediator roles of trait anxiety, hostility, and impulsivity in the association between childhood trauma and dissociation in male substance-dependent inpatients. Comprehensive psychiatry.

[CR32] Flores PJ (2004). Addiction as an attachment disorder.

[CR33] Folkman S, Moskowitz JT (2000). Positive affect and the other side of coping. American psychologist.

[CR34] Fossati A, Di Ceglie A, Acquarini E, Barratt ES (2001). Psychometric properties of an Italian version of the Barratt Impulsiveness Scale-11 (BIS-11) in nonclinical subjects. Journal of clinical psychology.

[CR35] Giannini M, Gori A, De Sanctis E, Schuldberg D (2011). Attachment in psychotherapy: Psychometric properties of the Psychological Treatment Inventory Attachment Styles Scale (PTI-ASS). Journal of Psychotherapy Integration.

[CR36] Goldstein AL, Haller S, Mackinnon SP, Stewart SH (2018). Attachment anxiety and avoidance, emotion disregulation, interpersonal difficulties and alcohol problems in emerging adulthood. Addiction Research & Theory.

[CR37] Gori A, Craparo G, Caretti V, Giannini M, Iraci-Sareri G, Bruschi A, Janiri L, Ponti L, Tani F (2016). Impulsivity, alexithymia and dissociation among pathological gamblers in different therapeutic settings: A multisaple comparison study. Psychiatry Research.

[CR38] Gori A, Giannini M, Schuldberg D (2015). PTI—Psychological Treatment Inventory.

[CR39] Grant JE, Odlaug BL, Mooney ME (2012). Telescoping phenomenon in pathological gambling: Association with gender and comorbidities. The Journal of nervous and mental disease.

[CR40] Green CL, Nahhas RW, Scoglio AA, Elman I (2016). Post-traumatic stress symptoms in pathological gambling: Potential evidence of anti-reward processes. Journal of behavioral addictions.

[CR41] Griffiths, M. D., Wood, R. T. A., Parke, J., & Parke, A. (2006). Dissociative states in problem gambling. *Current issues related to dissociation*, 27–37.

[CR42] Gross JJ, John OP (2003). Individual differences in two emotion regulation processes: implications for affect, relationships, and well-being. Journal of personality and social psychology.

[CR43] Hartmann M, Blaszczynski A (2018). The longitudinal relationships between psychiatric disorders and gambling disorders. International journal of mental health and addiction.

[CR44] Hasin DS, O’Brien CP, Auriacombe M, Borges G, Bucholz K, Budney A, Schuckit M (2013). DSM-5 criteria for substance use disorders: recommendations and rationale. American Journal of Psychiatry.

[CR45] Haw J (2017). Impulsivity predictors of problem gambling and impaired control. International Journal of Mental Health and Addiction.

[CR46] Hayes AF (2018). Introduction to mediation, moderation, and conditional process analysis second edition: A regression-based approach.

[CR47] Hayes AF, Preacher KJ (2014). Statistical mediation analysis with a multicategorical independent variable. British Journal of Mathematical and Statistical Psychology.

[CR48] Hazan C, Shaver PR (1994). Attachment as an organizational framework for research on close relationships. Psychological inquiry.

[CR49] Hodgins DC, Schopflocher DP, el-Guebaly, N., Casey, D. M., Smith, G. J., Williams, R. J., & Wood, R. T.  (2010). The association between childhood maltreatment and gambling problems in a community sample of adult men and women. Psychology of Addictive Behaviors.

[CR50] Iraci-Sareri G, Gori A (2012). Relazione tra Gioco d’Azzardo Patologico, alessitimia, sintomi dissociativi e impulsività: un confronto tra un gruppo di giocatori in trattamento e un gruppo di controllo [Relationship between pathological gambling, alexithymia, dissociative symptoms and impulsivity: a comparison between a group of players under treatment and a control one]. Italian Journal on Addiction.

[CR51] Jacobs DF (1986). A general theory of addictions: A new theoretical model. Journal of gambling behavior.

[CR52] James RJ, O’Malley C, Tunney RJ (2016). Loss of control as a discriminating factor between different latent classes of disordered gambling severity. Journal of Gambling Studies.

[CR53] John OP, Gross JJ (2004). Healthy and unhealthy emotion regulation: Personality processes, individual differences, and life span development. Journal of personality.

[CR54] Lam D (2007). An exploratory study of gambling motivations and their impact on the purchase frequencies of various gambling products. Psychology & Marketing.

[CR55] Lane W, Sacco P, Downton K, Luden E, Levy L, Tracy K (2016). Child maltreatment and problem gambling: A systematic review. Childe Abuse & Neglect.

[CR56] Lee HP, Chae PK, Lee HS, Kim YK (2007). The five-factor gambling motivation model. Psychiatry research.

[CR57] Leeman RF, Potenza MN (2012). Similarities and differences between pathological gambling and substance use disorders: a focus on impulsivity and compulsivity. Psychopharmacology (Berl).

[CR58] Lesieur, H. R. (2001). *Cluster analysis of types of inpatient pathological gamblers* (Doctoral dissertation, ProQuest Information & Learning).

[CR59] MacKillop J, Lisman SA, Weinstein A (2006). Psychometric validation of the Temptation and Restraint Inventory in two samples of college drinkers. Journal of Psychopathology and Behavioral Assessment.

[CR60] Maniaci G, Picone F, Dimarco T, Lipari A, Brancato A, Cannizzaro C (2015). Psychodiagnostic Assessment of Pathological Gamblers: A Focus on Personality Disorders, Clinical Syndromes and Alexithymia. International Journal of Mental Health & Addiction.

[CR61] Marchetti D, Verrocchio MC, Porcelli P (2019). Gambling Problems and Alexithymia: A Systematic Review. Brain sciences.

[CR62] Marchica LA, Mills DJ, Keough MT, Montreuil TC, Derevensky JL (2019). Emotion regulation in emerging adult gamblers and its mediating role with depressive symptomology. Journal of affective disorders.

[CR63] McCormick J, Delfabbro P, Denson L (2012). Psychological vulnerability and problem gambling: an application of Durand Jacobs’ general theory of addictions to electronic gaming machine playing in Australia. Journal of Gambling Studies.

[CR64] Melli G, Avallone E, Moulding R, Pinto A, Micheli E, Carraresi C (2015). Validation of the Italian version of the Yale-Brown Obsessive Compulsive Scale-Second Edition (Y-BOCS-II) in a clinical sample. Comprehensive psychiatry.

[CR65] Morris AS, Silk JS, Steinberg L, Myers SS, Robinson LR (2007). The role of the family context in the development of emotion regulation. Social development.

[CR66] Neighbors C, Lostutter TW, Cronce JM, Larimer ME (2002). Exploring college student gambling motivation. Journal of Gambling studies.

[CR67] Nijenhuis ER, Van der Hart O, Kruger K (2002). The psychometric characteristics of the Traumatic Experiences Checklist (TEC): First findings among psychiatric outpatients. Clinical Psychology & Psychotherapy: An International Journal of Theory & Practice.

[CR68] Oei TP, Gordon LM (2008). Psychosocial factors related to gambling abstinence and relapse in members of gamblers anonymous. Journal of Gambling Studies.

[CR69] Okechukwu CE (2019). Role of exercise in the treatment of gambling disorder. Nigerian Journal of Experimental and Clinical Biosciences.

[CR70] Pace, U., Zappulla, C., Di Maggio, R., Passanisi, A., & Craparo, G. (2015). Characteristics of regular gamblers in Italy: The role of control and emotion regulation. *Clinical Neuropsychiatry*, *12*(5).

[CR71] Patton JH, Stanford MS, Barratt ES (1995). Factor structure of the Barratt impulsiveness scale. Journal of clinical psychology.

[CR72] Perales JC, King DL, Navas JF, Schimmenti A, Sescousse G, Starcevic V, Billieux J (2020). Learning to lose control: A process-based account of behavioral addiction. Neuroscience & Biobehavioral Reviews.

[CR73] Potenza MN, Balodis IM, Derevensky J, Grant JE, Petry NM, Verdejo-Garcia A, Yip SW (2019). Gambling disorder. Nature Reviews Disease Primers.

[CR74] Power Y, Goodyear B, Crockford D (2012). Neural correlates of pathological gamblers preference for immediate rewards during the Iowa Gambling Task: an fMRI study. Journal of Gambling Studies.

[CR75] Quinn PD, Harden KP (2013). Differential changes in impulsivity and sensation seeking and the escalation of substance use from adolescence to early adulthood. Development and psychopathology.

[CR76] Rogier G, Velotti P (2018). Conceptualizing gambling disorder with the process model of emotion regulation. Journal of behavioral addictions.

[CR77] Schimmenti A (2016). Dissociative experiences and dissociative minds: Exploring a nomological network of dissociative functioning. Journal of Trauma & Dissociation.

[CR78] Schimmenti A (2018). The trauma factor: Examining the relationships among different types of trauma, dissociation, and psychopathology. Journal of Trauma & Dissociation.

[CR79] Schimmenti A, Passanisi A, Gervasi AM, Manzella S, Famà FI (2014). Insecure attachment attitudes in the onset of problematic Internet use among late adolescents. Child Psychiatry & Human Development.

[CR80] Schluter MG, Hodgins DC (2019). Dissociative experiences in Gambling Disorder. Current Addiction Reports.

[CR81] Shaffer HJ, Hall MN (2001). Updating and refining prevalence estimates of disordered gambling behaviour in the United States and Canada. Canadian Journal of Public Health.

[CR82] Sharpe L (2002). A reformulated cognitive–behavioral model of problem gambling: A biopsychosocial perspective. Clinical psychology review.

[CR83] Sherrer JF, Xian H, Kapp JM, Waterman B, Shah KR, Volberg R, Eisen SA (2007). Association between exposure to childhood and lifetime traumatic events and lifetime pathological gambling in a twin cohort. The journal of nervous and mental disease.

[CR84] Smith D, Harvey P, Battersby M, Pols R, Oakes J, Baigent M (2010). Treatment outcomes and predictors of drop out for problem gamblers in South Australia: a cohort study. Australian & New Zealand Journal of Psychiatry.

[CR85] Steel Z, Blaszczynski A (2002). Impulsivity, personality disorders and pathological gambling severity.

[CR86] Steward T, Mestre-Bach G, Fernández-Aranda F, Granero R, Perales JC, Navas JF, Menchón JM (2017). Delay discounting and impulsivity traits in young and older gambling disorder patients. Addictive Behaviors.

[CR87] Stewart SH (1996). Alcohol abuse in individuals exposed to trauma: a critical review. Psychological bulletin.

[CR88] Stewart SH, Zack M (2008). Development and psychometric evaluation of a three-dimensional Gambling Motives Questionnaire. Addiction.

[CR89] Storch EA, Rasmussen SA, Price LH, Larson MJ, Murphy TK, Goodman WK (2010). Development and psychometric evaluation of the Yale-Brown Obsessive-Compulsive Scale—Second Edition. Psychological assessment.

[CR90] Tang CSK, Lim MSM, Koh JM, Cheung FYL (2019). Emotion Dysregulation Mediating Associations Among Work Stress, Burnout, and Problem Gambling: A Serial Multiple Mediation Model. Journal of gambling studies.

[CR91] Taylor GJ, Bagby RM, Parker JDA (1997). Disorders of Affect Regulation: Alexithymia in Medical and Psychiatric Illness.

[CR92] Tice DM, Bratslavsky E, Baumeister RF (2001). Emotional distress regulation takes precedence over impulse control: If you feel bad, do it!. Journal of personality and social psychology.

[CR93] Wardell JD, Quilty LC, Hendershot CS, Bagby RM (2015). Motivational pathways from reward sensitivity and punishment sensitivity to gambling frequency and gambling-related problems. Psychology of Addictive Behaviors.

[CR94] Weatherly JN, Cookman ML (2014). Investigating several factors potentially related to endorsing gambling as an escape. Current Psychology.

[CR95] Widom CS, Weiler BL, Cottler LB (1999). Childhood victimization and drug abuse: a comparison of prospective and retrospective findings. Journal of consulting and clinical psychology.

[CR96] Williams AD, Grisham JR, Erskine A, Cassedy E (2012). Deficits in emotion regulation associated with pathological gambling. British Journal of Clinical Psychology.

[CR97] World Medical Association (2013). World Medical Association Declaration of Helsinki ethical principles for medical research involving human subjects. Journal of the American Medical Association.

[CR98] Zakiniaeiz Y, Cosgrove KP, Mazure CM, Potenza MN (2017). Does telescoping exist in male and female gamblers? Does it matter?. Frontiers in Psychology.

